# A Case of Fulminant West Nile Virus Encephalitis Presenting With Non-ST-Segment Elevation Myocardial Infarction (NSTEMI) and Diagnostic Discordance

**DOI:** 10.7759/cureus.103013

**Published:** 2026-02-05

**Authors:** Jayanjali Bodavula, Mindi S Garner

**Affiliations:** 1 Internal Medicine, Kansas City University College of Osteopathic Medicine, Joplin, USA; 2 Internal Medicine, Mercy Hospital Pittsburg, Pittsburg, USA

**Keywords:** blood donor screening, demand ischemia nstemi, diagnostic test discordance, neuroinvasive west nile virus, rapid neurologic decline, type 2 nstemi, viral encephalitis, west nile pcr sensitivity, west nile virus, west nile virus encephalopathy

## Abstract

West Nile virus (WNV) is a mosquito-borne flavivirus that typically causes mild febrile illness but can progress to neuroinvasive disease and even death, particularly in older adults with comorbidities. We describe a 77-year-old male with coronary artery disease, carotid artery disease, hypertension, and hyperlipidemia, who was notified that his recent blood donation had tested positive for WNV by nucleic acid testing (NAT) on initial screening, with confirmatory testing pending. Shortly thereafter, he developed a fever, confusion, dizziness, and worsening gait instability. On admission, he was febrile to 103.6°F, hypotensive at 97/65 mmHg, and noted to have monocytosis, elevated troponin, and electrocardiogram (ECG) abnormalities consistent with type II non-ST-segment elevation myocardial infarction (NSTEMI). He rapidly developed severe encephalopathy and acute hypoxemic respiratory failure. Lumbar puncture revealed elevated opening pressure (29 cm H₂O), neutrophil-predominant pleocytosis (265 WBC/µL), markedly elevated protein (149 mg/dL), and normal glucose. Despite findings consistent with viral encephalitis, cerebrospinal fluid (CSF) polymerase chain reaction (PCR) testing for WNV ribonucleic acid (RNA) was negative. Extensive testing for alternative bacterial, viral, and tickborne pathogens was also negative. Despite broad empiric antimicrobial and antiviral therapy, the patient deteriorated rapidly, was transitioned to comfort measures, and died within 48 hours of admission. This case highlights the diagnostic challenges of neuroinvasive WNV encephalitis, particularly in the setting of discordant or unavailable diagnostic testing. Molecular assays, such as CSF PCR for WNV RNA, have limited sensitivity and frequently yield false-negative results when performed outside the early viremic window. In contrast, serologic testing, such as CSF WNV immunoglobulin M (IgM) antibody, is more sensitive but may be limited by IgM cross-reactivity and clinical availability. In this patient, the prior positive blood donation NAT provided a rare early diagnostic clue, while the atypical NSTEMI presentation could have delayed recognition of neuroinvasive disease. Accurate diagnosis, therefore, requires synthesis of clinical presentation, epidemiologic exposure, and available diagnostic data, and clinicians should maintain a high index of suspicion for WNV encephalitis even when standard diagnostic tests are negative.

## Introduction

West Nile virus (WNV) is a mosquito-borne flavivirus that can cause disease ranging from asymptomatic infection to severe neuroinvasive disease [1]. WNV circulates primarily between birds and mosquitoes, with humans serving as incidental hosts [2]. Transmission to humans most commonly occurs through bites from infected *Culex *species mosquitoes, although cases linked to blood transfusion, organ transplantation, and vertical transmission have also been described [2]. In the United States, WNV infections occur predominantly during late summer and early fall, corresponding to peak mosquito season [1]. Individuals with frequent outdoor exposure, such as farmers, gardeners, and recreationalists, are at increased risk of infection during peak mosquito activity [3].

Most WNV infections are clinically silent, and only about 20% of infected patients develop a self-limited febrile illness characterized by fever, headache, myalgias, fatigue, and gastrointestinal symptoms [1,4]. Fewer than 1% progress to neuroinvasive disease, which may present as meningitis, encephalitis, or acute flaccid paralysis and can be associated with altered mental status, seizures, or focal neurologic deficits [1,4]. Severe outcomes are more common in older adults and individuals with cardiovascular or metabolic comorbidities. These patients experience higher rates of neuroinvasive disease, intensive care unit admission, and mortality [5].

Accurate diagnosis of neuroinvasive WNV infection remains challenging. Serologic testing, such as cerebrospinal fluid (CSF) WNV immunoglobulin M (IgM) antibody testing, is more sensitive and is the diagnostic test of choice for neuroinvasive disease according to the Infectious Diseases Society of America [6]. However, serologic testing may be limited by flavivirus cross-reactivity, which can complicate interpretation [6,7]. In contrast, molecular assays, such as CSF polymerase chain reaction (PCR) testing for WNV ribonucleic acid (RNA), are specific but have limited sensitivity [8]. False-negative results are common, particularly when testing occurs outside the early viremic phase of illness [8,9]. Therefore, physicians must rely on clinical presentation, epidemiologic exposure, and clinical judgment when establishing the diagnosis.

In this report, we describe a case of fulminant WNV encephalitis in which the patient’s recent blood donation tested positive on initial screening for WNV by nucleic acid testing (NAT), providing an early diagnostic clue despite a negative CSF PCR for WNV RNA. The case is further distinguished by its atypical presentation with type II non-ST-segment elevation myocardial infarction (NSTEMI), rapid neurologic and respiratory decline, and fatal progression within 48 hours of admission. This case underscores the diagnostic uncertainty physicians face when molecular testing is discordant with clinical findings and emphasizes the importance of integrating epidemiologic context with clinical presentation.

## Case presentation

A 77-year-old male with a history of coronary artery disease, carotid artery disease, hypertension, and hyperlipidemia presented with two days of weakness, fatigue, dizziness, staggering gait, and increasing confusion. He reported difficulty maintaining his balance and profound exhaustion, having slept nearly the entire previous day. Two weeks before admission, he had donated blood. Four days before symptom onset, he was notified by the American Red Cross that his donation had tested positive for WNV by NAT on initial screening, with confirmatory testing pending. The patient denied any travel history outside of the state of Kansas but reported frequent tick and mosquito exposure through farming work.

Upon admission, he was febrile at 103.6°F and hypotensive at 97/65 mmHg. He appeared fatigued, was slow to respond to questions, and required assistance from his wife for reporting history, although he was initially alert and oriented. An electrocardiogram (ECG) demonstrated sinus tachycardia with T-wave abnormalities and anterior/inferior Q waves. Troponin was elevated (32 ng/L; normal 15), consistent with type II NSTEMI, likely due to a supply-demand mismatch in the setting of systemic infection. Initial laboratory evaluation revealed elevated aspartate aminotransferase (AST) of 84 U/L (normal 0-50 U/L) and monocytosis on differential. Additional abnormalities included mild hyponatremia and hypocalcemia (Table 1).

**Table 1:** Progression of key laboratory parameters pH: potential hydrogen; PO2: partial pressure of oxygen; PCO2: partial pressure of carbon dioxide; HCO3: bicarbonate; N/A: not available

Parameter	Day 1	Day 2	Day 3	Reference Range
Temperature	103.6°F	101.8°F	99.9°F	98.6°F
Blood Pressure	97/65 mmHg	144/91 mmHg	116/72 mmHg	120/80 mmHg
White Blood Cells	8.45 K/μL	12.1 K/μL	16.1 K/μL	4.3 – 11.0 K/μL
Neutrophils	64.4%	79%	N/A	42 – 75%
Monocytes	17.3%	11%	N/A	12 – 44%
Sodium	133 mmol/L	138 mmol/L	130 mmol/L	136 – 145 mmol/L
Calcium	8.1 mmol/L	8 mmol/L	7.7 mmol/L	8.8 – 10.2 mmol/L
Aspartate Aminotransferase (AST)	84 U/L	193 U/L	322 U/L	0 – 50 U/L
Alanine Aminotransferase (ALT)	28 U/L	52 U/L	94 U/L	0 – 50 U/L
Troponin T	32 ng/L	35 ng/L	41 ng/L	≤ 15 ng/L
pH	N/A	7.52	N/A	7.37 – 7.43
PO_2_	N/A	70 mmHg	N/A	79 – 93 mmHg
PCO_2_	N/A	31 mmHg	N/A	35 – 45 mmHg
HCO_3_	N/A	25 mmol/L	N/A	23 – 27 mmol/L

Over the next two days, the patient remained febrile and developed progressive encephalopathy. On hospital day two, he became agitated and disoriented and developed hypoxemic respiratory failure with increased work of breathing. Arterial blood gas analysis obtained on day two revealed pH 7.52, PCO₂ 31 mmHg, and HCO₃ 25 mmol/L, consistent with respiratory alkalosis and hypoxemia (Table 1). By hospital day three, the patient had severe encephalopathy with hypercapnia and hypoxia. He was extremely lethargic, nonverbal, not following commands, and minimally responsive to noxious stimulation. The neurologic exam was notable for pinpoint pupils, wandering eye movements, and oral secretions, though seizure activity was not observed. Oxygen saturation dropped into the 70s despite being on a 6L nasal cannula. Laboratory evaluation showed increasing troponin (41 ng/L), leukocytosis (16.1 K/µL), and worsening transaminitis (AST of 322 U/L and alanine aminotransferase (ALT) of 94 U/L) (Table 1).

Lumbar puncture revealed an elevated opening pressure (29 cm H₂O), CSF pleocytosis (265 WBC/µL; 59% neutrophils, 41% monocytes), elevated protein (149 mg/dL), and normal glucose. CSF gram stain and culture showed no organisms (Table 2). A multiplex CSF meningitis pathogen RNA PCR panel, testing for various bacterial, fungal, and viral pathogens, including WNV RNA, came back negative. Blood tickborne deoxyribonucleic acid (DNA) PCR panels were also negative. Blood cultures showed no growth after five days (Table 3).

**Table 2:** Cerebrospinal fluid (CSF) findings from lumbar puncture

CSF Parameter	Patient’s CSF Value	Reference Range
Opening Pressure	29 cm H2O	6-25 cm H2O
Appearance	Clear	Clear
Color	Colorless	Colorless
White Blood Cells	265 /μL	0 – 5 /μL
Red Blood Cells	50 /μL	0 – 5 /μL
Neutrophils	59%	0 – 2%
Monocytes	41%	3 – 37%
Glucose	64 mg/dL	40 – 70 mg/dL
Protein	149 mg/dL	15 – 45 mg/dL
Gram Stain and Culture	No organisms, No growth	No organisms, No growth

**Table 3:** Infectious disease diagnostic work-up and results NAT: nucleic acid testing; CSF: cerebral spinal fluid; RNA: ribonucleic acid; PCR: polymerase chain reaction; DNA: deoxyribonucleic acid CSF Meningitis PCR Panel checked for: *Escherichia coli* K1, *Haemophilus influenzae*, *Listeria monocytogenes*, *Neisseria meningitidis*, *Streptococcus agalactiae*, *Streptococcus pneumoniae*, *Cytomegalovirus*, *Enterovirus*, Herpes simplex virus 1 and 2, Human herpesvirus 6, Human parechovirus, Varicella zoster virus, *Cryptococcus neoformans*/*gattii* Blood Tickborne PCR Panel checked for: *Anaplasma Phagoctyophilum*, *Babesia Microti*, *Borrelia sp.*, *Borrelia Miyamotoi*, *Ehrlichia Chaffeensis*, *Ehrlichia Ewingii*

Workup	Patient’s Result	Reference Range
Blood Donation West Nile Virus NAT	Positive	Negative
CSF West Nile Virus RNA PCR	Not detected	Not detected
CSF Meningitis Pathogen RNA PCR Panel	Not detected	Not detected
Blood Tickborne DNA PCR Panel	Not detected	Not detected
Blood Culture	No growth after 5 days	No growth after 5 days

Initial imaging and cardiac evaluation did not reveal an alternative explanation for the patient’s rapid neurologic decline. Transthoracic echocardiogram demonstrated normal left and right ventricular size and systolic function, with a preserved left ventricular ejection fraction of 60% and no wall motion abnormalities (Video 1). Despite empirical broad-spectrum antibacterial, antiviral, and tickborne coverage (ceftriaxone, vancomycin, ampicillin, acyclovir, and doxycycline), the patient declined rapidly. Given his poor prognosis, the family elected do-not-resuscitate status and transitioned to comfort-focused care. The patient died on hospital day three, less than 48 hours after admission. The chronological order of events is summarized in Table 4.

**Video 1:** Transthoracic echocardiogram showing normal left and right ventricular size and systolic function, with a preserved left ventricular ejection fraction of 60% and no wall motion abnormalities

**Table 4:** Clinical timeline of events and hospital course WNV: West Nile virus, NAT: nucleic acid testing, NSTEMI: non-ST-segment elevation myocardial infarction, ICU: intensive care unit, LP: lumbar puncture, CSF: cerebrospinal fluid, PCR: polymerase chain reaction, RNA: ribonucleic acid

Hospital Day (in relation to the day of admission)	Event/Progress
2 weeks prior to admission	Donated blood.
4 days prior to admission	Received letter from American Red Cross that blood donation tested positive for WNV on initial screening with NAT and confirmatory testing pending.
2 days prior to admission	Started experiencing weakness, fatigue, dizziness, confusion, staggering gait, and difficulty maintaining balance.
Day 1	Presented with high fever, hypotension, lethargy, and NSTEMI. Transferred to ICU.
Day 2	Worsening encephalopathy. LP performed with elevated opening pressure, pleocytosis, and high protein. CSF PCR for WNV RNA negative. Troponins further increased. Hypoxemic respiratory failure developed.
Day 3	Rapid decline: severe encephalopathy, minimally responsive to noxious stimuli, respiratory failure. Transitioned to comfort care. Died within 48 hours of admission.

## Discussion

Diagnosis of neuroinvasive WNV infection remains challenging and often requires integration of clinical presentation, epidemiologic exposure, and clinical judgment rather than reliance on a single diagnostic test. Currently, serologic CSF WNV IgM antibody testing is widely regarded as the diagnostic test of choice for neuroinvasive disease and is recommended by the Infectious Diseases Society of America [6]. Although highly sensitive, estimated at 95% in CSF, this test has certain limitations [10].

One limitation is IgM antibody cross-reactivity between flaviviruses within the same serocomplex, including dengue virus and St. Louis encephalitis virus, due to shared antigenic features [7,10]. As a result, both species-specific and cross-reactive antibodies are produced during flavivirus infections [7]. In endemic regions where multiple flaviviruses circulate, individuals accumulate cross-reactive antibodies from previous exposures that can persist in the CSF, making it difficult to differentiate between acute and past infections [7,10]. This diagnostic challenge is particularly relevant in populations with repeated environmental exposure, such as farmers [3,7]. Furthermore, access to serologic testing may be limited in certain clinical settings, particularly when testing requires sending out to reference or public health laboratories [7]. In such circumstances, as in our case, where serologic testing is unavailable, clinicians may need to rely on molecular assays instead.

Consistent with prior studies, molecular detection of WNV RNA by PCR is specific but has limited sensitivity, estimated at 55% in CSF [8,9]. This is because viral RNA in CSF is typically present at low, transient levels and is often cleared by the host immune response prior to the onset of neurologic symptoms [9]. Prior studies have demonstrated that PCR sensitivity is highest during the early viremic phase and declines substantially once neuroinvasive manifestations emerge [8,9]. Consequently, false-negative CSF PCR results are common, especially when viral loads are low or when testing occurs outside the early viremic window [8].

These limitations of molecular assays are consistent with the findings in our patient, whose CSF PCR for WNV RNA was negative despite a classic presentation of viral encephalitis. Specifically, the patient exhibited febrile prodrome, rapid progression to encephalopathy, and CSF pleocytosis with elevated protein and normal glucose. His fulminant deterioration to respiratory failure and death within 48 hours further supported a viral etiology rather than metabolic or bacterial causes. Despite this, diagnostic molecular testing, including CSF PCR for WNV RNA, multiplex meningitis pathogen panel, and blood tickborne panel, was all negative. This diagnostic discordance, coupled with the unavailable serologic testing, could have obscured the diagnosis. However, the patient’s prior positive blood donation NAT provided a crucial diagnostic clue supporting WNV as the most likely etiology. Prior studies of blood donor screening programs have demonstrated that NAT can detect low-level viremia during early WNV infection, often several days before symptom onset and prior to the development of detectable antibody responses [2]. Therefore, blood donation NAT results may represent the earliest available diagnostic evidence of infection [2]. Nonetheless, such information is rarely available to clinicians in real time. Physicians must therefore maintain a high index of suspicion for WNV encephalitis when the clinical presentation and epidemiologic context are consistent, even in the absence of confirmatory molecular or serologic results.

In addition to diagnostic uncertainty, this patient also presented with troponin elevation and ECG changes consistent with type II NSTEMI. This is an unusual manifestation that could have suggested a primary cardiac process rather than an infectious or neurologic one. Although cardiac involvement in WNV is rare, prior cases of WNV presenting with myocarditis, arrhythmias, and troponin elevation have been reported [11,12]. These manifestations are thought to be a result of systemic inflammation, cytokine-mediated myocardial injury, or direct viral involvement of cardiac tissue [12]. In our patient, the absence of echocardiographic wall abnormalities, combined with systemic inflammation and hypoxemia, suggests myocardial injury due to demand ischemia rather than a primary cardiac pathology. While the prior NAT result ultimately clarified the diagnosis, the atypical NSTEMI presentation had the potential to misdirect clinical reasoning. This highlights the importance of recognizing that WNV can manifest with atypical systemic features that can complicate the diagnostic process.

Taken together, this case expands the existing literature by demonstrating how neuroinvasive WNV infection may present with atypical systemic manifestations and discordant diagnostic studies. Consistent with prior studies, our findings highlight that no single laboratory test is sufficient to diagnose neuroinvasive WNV and that diagnosis often requires synthesis of clinical presentation, epidemiologic exposure, clinical judgment, and available diagnostic data [1,4,5]. Early clinical suspicion remains critical, particularly in endemic regions during peak transmission seasons.

## Conclusions

This case illustrates the diagnostic challenges of neuroinvasive WNV encephalitis, particularly in the setting of discordant or unavailable diagnostic testing. Despite a classic clinical presentation and CSF findings consistent with viral encephalitis, molecular diagnostic studies, including CSF PCR for WNV RNA, were negative. This finding was consistent with the known limitations of molecular PCR sensitivity outside the early viremic window. Although the patient’s prior positive blood donation NAT provided a critical diagnostic clue, such information is rarely available to clinicians in real time. Physicians must therefore consider WNV encephalitis when the clinical presentation and epidemiologic context are consistent, even in the absence of confirmatory diagnostic tests. Additionally, the patient’s atypical presentation with type II NSTEMI demonstrates how systemic manifestations can confound clinical reasoning and delay recognition of the underlying etiology. Overall, this case reinforces that no single test is sufficient to diagnose neuroinvasive WNV. Accurate diagnosis instead requires synthesis of compatible clinical features, epidemiologic exposure, and clinical judgement, even when standard diagnostic tests are negative. Maintaining a high index of suspicion remains essential, particularly in endemic regions during peak transmission seasons.
